# Fission Yeast 26S Proteasome Mutants Are Multi-Drug Resistant Due to Stabilization of the Pap1 Transcription Factor

**DOI:** 10.1371/journal.pone.0050796

**Published:** 2012-11-27

**Authors:** Mary Penney, Itaru Samejima, Caroline R. Wilkinson, Christopher J. McInerny, Søs G. Mathiassen, Mairi Wallace, Takashi Toda, Rasmus Hartmann-Petersen, Colin Gordon

**Affiliations:** 1 Medical Research Council, Human Genetics Unit, Western General Hospital, Edinburgh, United Kingdom; 2 Cell Regulation Group, Paterson Institute for Cancer Research, University of Manchester, Manchester, United Kingdom; 3 Division of Molecular and Cellular Biology, School of Life Sciences, College of Medical, Veterinary and Life Sciences, University of Glasgow, Glasgow, United Kingdom; 4 Laboratory of Cell Regulation, Cancer Research UK, London Research Institute, Lincoln's Inn Fields Laboratories, London, United Kingdom; 5 Department of Biology, University of Copenhagen, Copenhagen, Denmark; University of Minnesota, United States of America

## Abstract

Here we report the result of a genetic screen for mutants resistant to the microtubule poison methyl benzimidazol-2-yl carbamate (MBC) that were also temperature sensitive for growth. In total the isolated mutants were distributed in ten complementation groups. Cloning experiments revealed that most of the mutants were in essential genes encoding various 26S proteasome subunits. We found that the proteasome mutants are multi-drug resistant due to stabilization of the stress-activated transcription factor Pap1. We show that the ubiquitylation and ultimately the degradation of Pap1 depend on the Rhp6/Ubc2 E2 ubiquitin conjugating enzyme and the Ubr1 E3 ubiquitin-protein ligase. Accordingly, mutants lacking Rhp6 or Ubr1 display drug-resistant phenotypes.

## Introduction

Intracellular protein degradation is a regulated process that maintains cellular homeostasis [1]. However, selective destruction of regulatory proteins also provides an important control mechanism for quickly and irreversibly eliminating signalling proteins such as transcription factors [1]. Intracellular protein degradation is therefore relevant for most cellular and physiological functions including apoptosis, cell cycle progression, differentiation and DNA repair [1], and also partakes in cellular stress responses [2].

In eukaryotic cells the major degradation pathway for intracellular proteins is via the ubiquitin-proteasome system (UPS) [3] [1] [4]. This system relies on a cascade of three enzymes termed E1, E2 and E3 that conjugate the small protein ubiquitin to specific target proteins [3] [5]. Subsequently, the proteins, which have been marked with ubiquitin, are targeted to the 26S proteasome, a large proteolytic particle found in the nucleus and cytosol of all eukaryotic cells [4]. At the 26S proteasome the ubiquitin chains are released while the substrate is degraded.

The 26S proteasome is composed of two subcomplexes, the proteolytically active 20S core particle and 19S regulatory complexes that bind to one or both ends of the 20S particle [4]. Structurally the 20S core is built from 28 subunits, arranged as four stacked heptameric rings, forming a cylindrical structure [6]. The two outer rings each contain seven different α subunits (α_1_- α_7_) and the two inner rings each contain seven different β subunits (β_1_– β_7_), forming an overall α_1–7_β_1–7_β_1–7_α_1–7_ structure [6]. Some of the β subunits are threonine-type proteases that expose their active sites towards a central chamber inside the 20S cylinder [6].

The 19S regulatory complex is an asymmetric particle composed of about 19 different subunits distributed between two subcomplexes called the base and the lid [4]. Some of these subunits are responsible for binding ubiquitylated substrates, while others are involved in recycling ubiquitin, by cleaving the ubiquitin moieties from the substrate during degradation. The 19S particle also contains six different ATPase subunits that function in unfolding and translocation of the protein substrates into the 20S cylinder [7]–[8].

In the fission yeast *Schizosaccharomyces pombe* a number of mutants have been isolated by their ability to be resistant to the mitotic poison methyl benzimidazol-2-yl carbamate (MBC) and also be temperature sensitive for growth, and were named *mts* for “MBC resistant and temperature sensitive”. Most of the *mts* mutants identified by this screen were found to be in different subunits of the 26S proteasome [9]–[12]. Although the 26S proteasome, through degradation of various substrates, is involved in multiple cellular pathways, the reason for the enrichment of 26S proteasome mutants in the screen has remained elusive.

The *S. pombe* homolog of the human AP-1 transcription factor, Pap1, is one of the major stress activated transcription factors in fission yeast [13]. Overexpression of Pap1 results in resistance to a number of different drugs such as staurosporine [14] and brefeldin A [15]. Conversely, mutants lacking Pap1 are hypersensitive to drugs such as caffeine [16].

Here, we characterize six novel *mts* mutants. Five of these mutants are in subunits of the 26S proteasome, while one is in the nuclear export receptor, Crm1. We show that the proteasome mutants are multi-drug resistant. This phenotype depends on the Pap1 transcription factor that is degraded by the ubiquitin pathway, but stabilized in the proteasome mutants. Finally, we also show that the Rhp6/Ubc2, E2 ubiquitin conjugating enzyme and the Ubr1 E3 ubiquitin-protein ligase are responsible for ubiquitylation of Pap1, and targeting Pap1 for degradation by the 26S proteasome.

## Materials and Methods

### S. pombe Strains, Techniques and Reagents

The *S. pombe* strains used in this study (Table 1) are derivatives of the wild type heterothallic strains *972h^−^* and *975h^+^*. Standard genetic methods and media were used and *S. pombe* transformations were performed using the lithium acetate procedure [17]. The PCR mutagenesis was performed according to a previously published procedure [18]. Methyl benzimidazol-2-yl carbamate (MBC) was purchased from Sigma.

**Table 1:** Fission yeast strains used in this study.

*Strain*	*Genotype*	*Reference*
*wild type*	*leu1-32 ura4-D18 h-*	Laboratory stock
*pap1*Δ	*pap1::ura4 leu1-32 ura4-D18 h-*	[14]
*cdc25.M35*	*cdc25.M35 leu1-32 h-*	[44]
*mts1-1*	*mts1-1 leu1-32 ura4-D18 h-*	This study
*mts2-1*	*mts2-1 leu1-32 ura4-D18 h-*	[9]
*mts3-1*	*mts3-1 leu1-32 ura4-D18 h-*	[10]
*mts4-1*	*mts4-1 leu1-32 ura4-D18 h-*	[11]
*mts5-1*	*mts5-1 (pad1-1) leu1-32 ura4-D18 h-*	[12]
*mts6-1*	*mts6-1 leu1-32 ura4-D18 h-*	This study
*mts7-1*	*mts7-1 leu1-32 ura4-D18 h-*	This study
*mts8-1*	*mts8-1 leu1-32 ura4-D18 h-*	This study
*mts9-1*	*mts9-1 leu1-32 ura4-D18 h-*	This study
*mts10-1*	*mts10-1(crm1) leu1-32 ura4-D18 h-*	This study
*ubc1*Δ	*ubc1::ura4 leu1-32 ura4-D18 h-*	[45]
*rhp6*Δ	*rhp6(ubc2)::ura4 leu1-32 ura4-D18 h-*	[45]
*ubc3*Δ	*ubc3::ura4 leu1-32 ura4-D18 h-*	[45]
*ubc4-1*	*ubcP1 leu1-32 ura4-D18 h-*	[46]
*ubc6*Δ	*ubc6::ura4 leu1-32 ura4-D18 h-*	[45]
*ubc8*Δ	*ubc8::ura4 leu1-32 ura4-D18 h-*	This study
*ubc11-1*	*ubcP4 leu1-32 ura4-D18 h-*	[46]
*ubc13*Δ	*ubc13::ura4 leu1-32 ura4-D18 h-*	[47]
*ubc14*Δ	*ubc14::ura4 leu1-32 ura4-D18 h-*	This study
*ubc15*Δ	*ubc15::ura4 leu1-32 ura4-D18 h-*	[48]
*ubc16*Δ	*ubc16::HYG leu1-32 ura4-D18 h-*	This study
*rhp18*Δ	*rhp18::ura4 leu1-32 ura4-D18 h-*	[45]
*ubr1*Δ	*ubr1::ura4 leu1-32 ura4-D18 h-*	[45]
*ubr11*Δ	*ubr11::ura4 leu1-32 ura4-D18 h-*	[45]
*cdc8-27*	*cdc8-27 leu1-32, h-*	[49]
*cdc13-117*	*cdc13-117 leu1-32*	[50]
*sep1*Δ	*sep1::G418 ura4-D18 leu1-32 h-*	[51]
*cdt2*Δ	*cdt2::G418 ura4-D18 leu1-32 h-*	[52]
*txl1*Δ	*txl1::NAT leu1-32 ura4-D18 h-*	[53]

### Antibodies

The antibody to tubulin was the TAT1 monoclonal (Sigma). The antibody to actin was from GE Healthcare. The antibody to GFP was purchased from Roche. The antibodies to Obr1 (p25) and Pap1 have been described previously [19]. The antibody to Mts4 has been described previously [11]. The antibody to the 20S alpha subunits was the monoclonal MCP231 from Enzo Life Sciences. Secondary antibodies were from Dako. All antibodies were used in 1∶1000 dilutions.

### Plasmids and Purification

The expression constructs used here were wild type cDNA encoding Pap1 and ubiquitin N-terminally tagged with GFP and 6His, respectively, subcloned to the pREP41 *S. pombe* expression vector. 6His-tagged ubiquitin was purified on Ni^2+^-NTA agarose beads (Qiagen) under denaturing conditions in 8 M urea as described by the manufacturer.

The *S. pombe* cDNA library was generously supplied by Prof. Peter A. Fantes (Edinburgh, UK).

### Protein Degradation Assays

Protein degradation kinetics were determined by SDS-PAGE and blotting of extracts prepared from cultures treated with cycloheximide (CHX), as described previously [20].

## Results

### Isolation of the mts Mutants

We carried out a screen to isolate mutants that were resistant to the mitotic poison methyl benzimidazol-2-yl carbamate (MBC) and also temperature sensitive for growth. In total 24 mutants were obtained. Crossing them to each other demonstrated that the mutants lay in 10 complementation groups (*mts1-mts10*) (Table 2). Genetic analyses showed that in each case the temperature sensitive and drug resistant phenotypes co-segregated demonstrating that the same mutation was responsible for both phenotypes.

**Table 2:** The mts complementation groups.

Mtsgroup	No. ofalleles	Encoded protein	Function
mts1	3	Rpn9	19S lid proteasome subunit,
mts2	4	Rpt2	19S base proteasome subunit
mts3	1	Rpn12	19S lid proteasome subunit
mts4	6	Rpn1	19S base proteasome subunit
mts5	1	Rpn11/Pad1	19S lid proteasome subunit
mts6	2	β2/Pup1	20S proteasome subunit
mts7	1	α4/Pre6	20S proteasome subunit
mts8	1	β1/Pre3	20S proteasome subunit
mts9	1	β7/Pre4	20S proteasome subunit
mts10	4	Crm1	Nuclear export receptor

### Cloning of mts1, mts6, mts7, mts8, mts9 and mts10

Previous published work identified *mts2, mts3, mts4* and *mts5/pad1* mutants to be in different subunits of the 26S proteasome [9]–[12]. All four mutant strains were in different subunits of the 19S regulatory complex. The *mts2* ^+^ gene encodes the Rpt2 base ATPase subunit, *mts3^+^* the Rpn12 lid subunit, *mts4^+^* the base non-ATPase Rpn1 and *mts5^+^* the lid Rpn11/Pad1 deubiquitylating subunit (Table 2). To identify the genes encoding the *mts1, mts6, mts7, mts8, mts9* and *mts10* mutants the temperature sensitive phenotype of each mutant strain was rescued by transformation with a fission yeast cDNA library in an *S. pombe* expression vector. After isolating and sequencing plasmids it was observed that five of the mutant strains (*mts1, mts6, mts7*, *mts8* and *mts9*) were rescued by cDNAs that encoded different subunits of the 26S proteasome. The *mts1^+^* gene was found to encode the lid Rpn9 subunit. The *mts6, mts7, mts8* and *mts9* were rescued by cDNA encoding the 20S proteasome core subunits β2, α4, β1 and β7, respectively (Table 2). Finally, the cDNA that complemented the *mts10* temperature sensitive phenotype encoded the nuclear export protein Crm1 (Table 2). To demonstrate that the cloned cDNAs encoded the authentic genes and not extragenic suppressors, each of the *mts* strains were crossed to mutants in closely linked genes: *cdt2* for *mts1*, *cdc8* for *mts6, cdc13* for *mts7, sep1* for *mts8, txl1* for *mts9* and *sep1* for *mts10.* In each case strong linkage was observed indicating that the temperature sensitive mutations were in the authentic genes (data not shown). In all cases deleting the *mts* genes resulted in lethality, revealing that the mutants were all conditional mutant alleles of essential genes. As nine out of ten genes encoded subunits of the 26S proteasome (Table 2) this raised the important question of why the screen was so biased for isolation of mutants in different subunits of the 26S proteasome.

### The mts Mutants are Multi-drug Resistant

**Figure 1:**
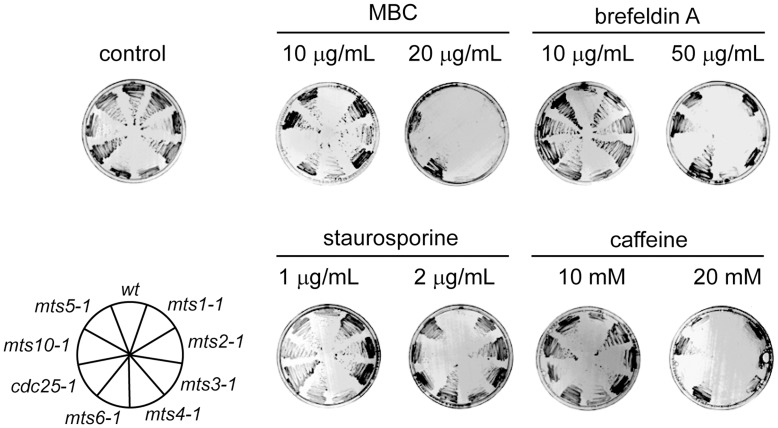
The mts mutants are multi-drug resistant. The indicated yeast strains (lower left panel) were streaked onto solid medium containing MBC, brefeldin A, staurosporine or caffeine at the shown concentrations and incubated for 48 hours at room temperature. On the control medium lacking drugs (upper left panel) all the strains grew. When the indicated drugs were added to the media the growth of wild type cells was compromised, while the *mts* mutants displayed resistance.

The *mts* mutants were isolated on account of their resistance to the mitotic poison MBC. We were interested to ask if the mutants were resistant to other drugs. When the *mts* strains were streaked on complete media containing the drugs brefeldin A, staurosporine or caffeine at the permissive temperature of 25°C, the *mts* mutants were found to be more resistant than wild type cells (Fig. 1). To show that this was not an effect of the temperature sensitivity of the mutants, a temperature sensitive mutant in *cdc25,* a gene encoding a cell cycle regulated phosphatase, was included as a control. The *mts* mutants all showed the same spectrum of resistance although the level of resistance varied with the different mutants (Fig. 1). The *cdc25* mutant was moderately resistant to staurosporine, but did not display the multi-drug resistant phenotype of the *mts* mutants (Fig. 1). These data confirm that the *mts* mutants are multi-drug resistant.

### The Pap1 Protein is Stabilized in the mts Mutants at the Permissive Temperature

**Figure 2:**
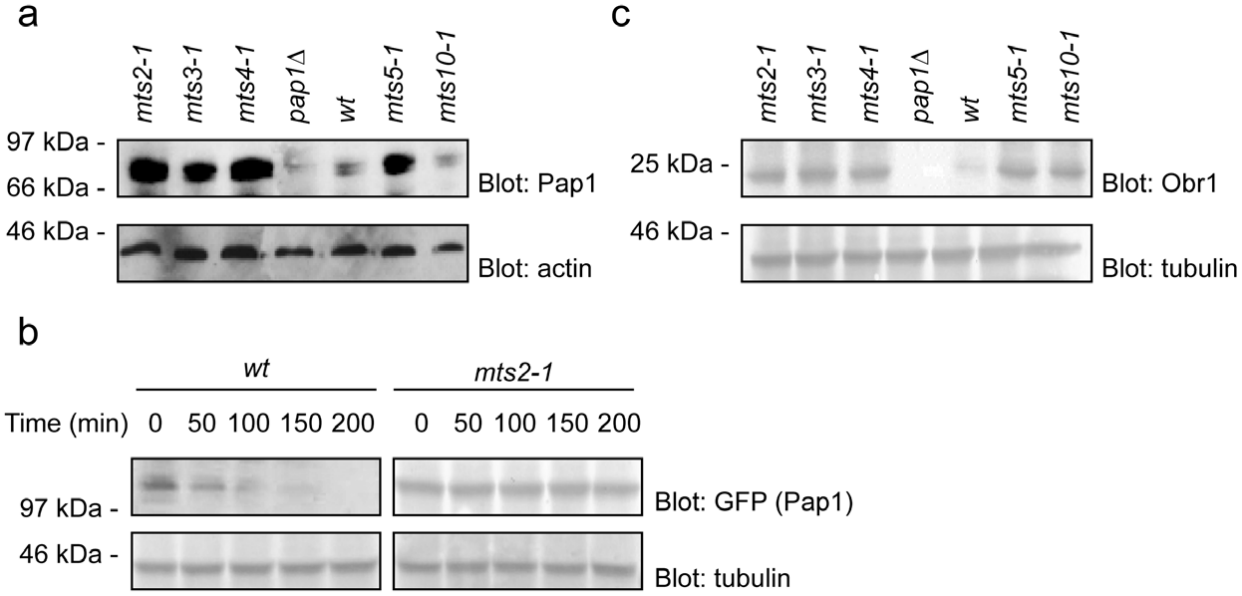
Stabilization of Pap1 in the mts mutants leads increased obr1^+^ expression. (a) To compare the steady state levels of Pap1 cell extracts of the indicated strains were prepared and analyzed by SDS-PAGE and Western blotting using antibodies to Pap1. Actin served as a loading control. Compared to wild type cells, the Pap1 levels were increased in the proteasome mutants, but not in the *mts10-1* (*crm1*) mutant. A *pap1*Δ mutant was included as a control. (b) The degradation kinetics of GFP-tagged Pap1 was followed by blotting of wild type (wt) and *mts2-1* cultures treated with cycloheximide (CHX). α-tubulin served as a loading control. In wild type cells Pap1 was rapidly degraded with a half-life of about 50 minutes. In the *mts2-1* background Pap1 was stabilized (c) To compare the steady state levels of the Pap1 target Obr1 cell extracts of the indicated strains were prepared and analyzed by SDS-PAGE and Western blotting using antibodies to Obr1. Tubulin served as a loading control. Compared to wild type cells, the Obr1 levels were increased in the proteasome mutants and, as expected, in the *mts10-1* (*crm1*) mutant. No Obr1 was detected in the *pap1*Δ mutant.

The multi-drug resistant phenotype has been observed before for cells in which the Pap1 transcription factor is overexpressed [14]–[15]. Indeed temperature and cold sensitive alleles of the *crm1* (*mts10*) gene display multi-drug resistance that depends on the presence of a wild type *pap1^+^* gene [19]. This raised the possibility that the proteasome mutants were multi-drug resistant due to the Pap1 protein being a substrate of the proteasome. Hence, Pap1 could be stabilized in the proteasome mutants because proteolysis is defective in these strains. To test this hypothesis the steady state levels of the Pap1 protein were determined in the *mts2-1, mts3-1, mts4-1, mts5-1* and wild type cells by Western blot analysis using an antibody to the Pap1 protein. The Pap1 protein was significantly more abundant in extracts prepared from the *mts* mutants compared to those prepared from wild type cells (Fig. 2a). In contrast, the level of Pap1 protein in the *crm1/mts10* mutant strain was unchanged compared to wild type (Fig. 2a). This is consistent with a different mechanism for multi-drug resistance in the *crm1-1* (*mts10-1*) strain compared to the proteasome mutants. Thus, in the *crm1-1* strain nuclear export of the Pap1 protein is impaired leading to drug resistance, as previously described [13].

To further demonstrate that the Pap1 protein was stabilized in the proteasome *mts* mutants, the degradation of Pap1 was followed in cultures treated with the translation inhibitor cycloheximide. The Pap1 protein was fused to GFP and expressed in wild type cells and the *mts2-1* proteasome mutant strain at the restrictive temperature of 36°C in the presence of cycloheximide. Extracts were prepared at intervals and analyzed by SDS-PAGE and blotting using an antibody to GFP. As expected the Pap1-GFP fusion protein was stabilized in the proteasome mutant compared to wild type cells (Fig. 2b).

### The mts Mutants Contain Elevated Levels of the Pap1 Target Obr1

Although the Pap1 protein is stabilized in the *mts* mutants, it is important to demonstrate that this increased amount of Pap1 protein augments the gene expression of Pap1 target genes. The *obr1^+^* gene contains a consensus Pap1 DNA binding motif in its promoter and has been shown to be transcribed specifically by Pap1 [19]. To show that the increased amount of Pap1 protein resulted in an increase in *obr1* ^+^ expression, extracts were prepared from the *mts* strains and analysed by Western blotting with antibodies to Obr1. Indeed, the level of the Obr1 was significantly upregulated in the *mts* mutants as compared to wild type cells (Fig. 2c). This demonstrates that the increased amount of Pap1 protein observed in the *mts* mutants results in an increase in Pap1-mediated gene expression.

### The Pap1 Protein is the Cause of the Multi-drug Resistant Phenotype of the mts Mutants

**Figure 3:**
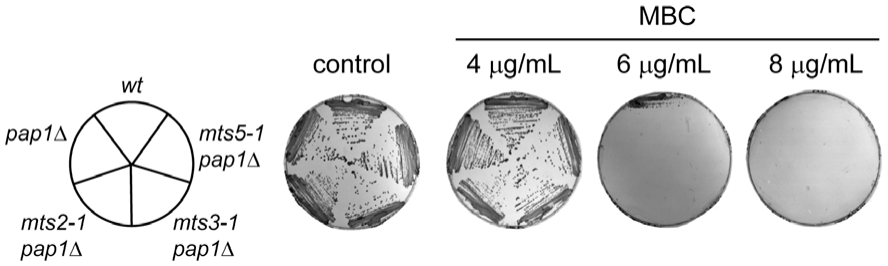
The multi-drug resistance of the mts mutants depends on Pap1. The indicated yeast strains (left panel) were streaked onto solid medium containing MBC at the shown concentrations and incubated for 48 hours at room temperature. On the control medium lacking drugs all the strains grew. In the presence of 6 µg/mL MBC some growth of the wild type was still apparent, while no growth of the other strains was observed.

If the observed stabilization of the Pap1 protein is the true cause of the multi-drug resistance observed in the *mts* mutants then this phenotype should be lost in strains which have been deleted for the *pap1* ^+^ gene. Cells lacking Pap1 are viable although they are stress sensitive [21]. Therefore the *pap1* null strain was crossed to each of the *mts2-1, mts3-1* and *mts5-1* mutants to construct *pap1*Δ*mts* double mutants. The MBC drug resistance phenotype was then investigated for each of the double mutant strains. The *pap1*Δ*mts* double mutants were all as sensitive to MBC as the *pap1*Δ strain, and were now more sensitive to MBC than wild type cells (Fig. 3). Similar results were obtained with brefeldin A, staurosporine and caffeine (data not shown). This strongly suggests that the observed multi-drug resistance phenotype observed in the *mts* mutants is the result of the stabilization of Pap1, and that the Pap1 protein is the primary target whose misregulation results in a multi-drug resistant phenotype in the proteasome mutants.

### The Pap1 Protein is Polyubiquitylated

**Figure 4:**
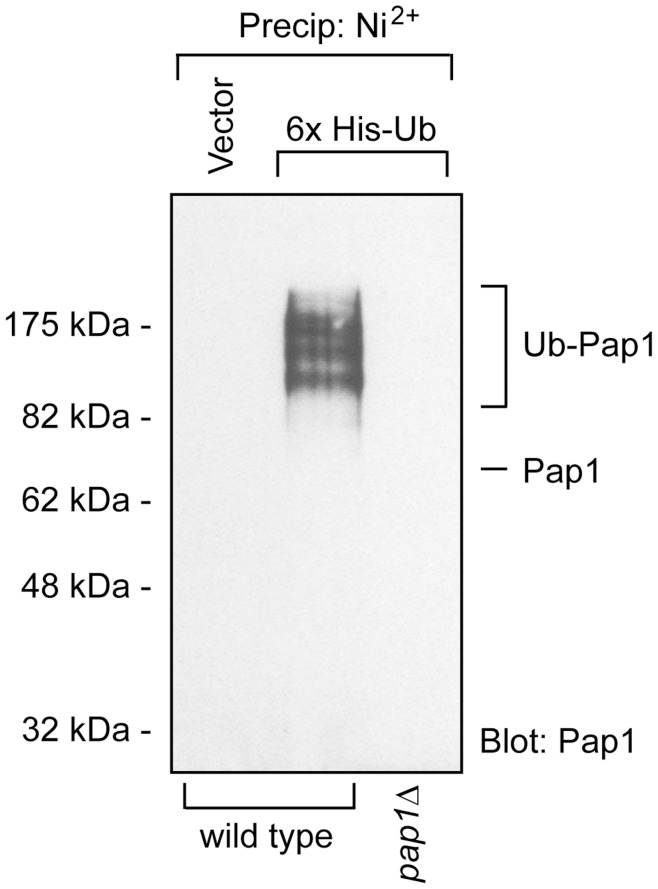
Pap1 is ubiquitylated. To determine the ubiquitylation status of Pap1, wild type cells and as a control a *pap1*Δ strain were transformed to express 6His-tagged ubiquitin. The 6His-tagged ubiquitin was then precipitated using Ni^2+^ agarose under denaturing conditions in 8 M urea. The precipitates were then washed three times in a denaturing buffer and analyzed by Western blotting using antibodies to Pap1. Prior to precipitation, the protein concentrations were determined and normalized. In total 5 mg cell protein was used for each precipitation. Ubiquitylated species of Pap1 were detected in wild type cells expressing the 6His-tagged ubiquitin, but not in the *pap1*Δ strain or in the vector control.

If the Pap1 transcription factor is a target of the 26S proteasome one would expect that it should be polyubiquitylated. To test this, 6His-tagged ubiquitin was expressed in *S. pombe* cells and purified on a nickel resin. The tagged ubiquitin conjugates were resolved by SDS-PAGE and analysed by Western blotting using antibodies to Pap1. Indeed we found that Pap1 was heavily ubiquitylated (Fig. 4).

### Rhp6/Ubc2 Functions as the E2 Ubiquitin-conjugating Enzyme for Pap1

**Figure 5:**
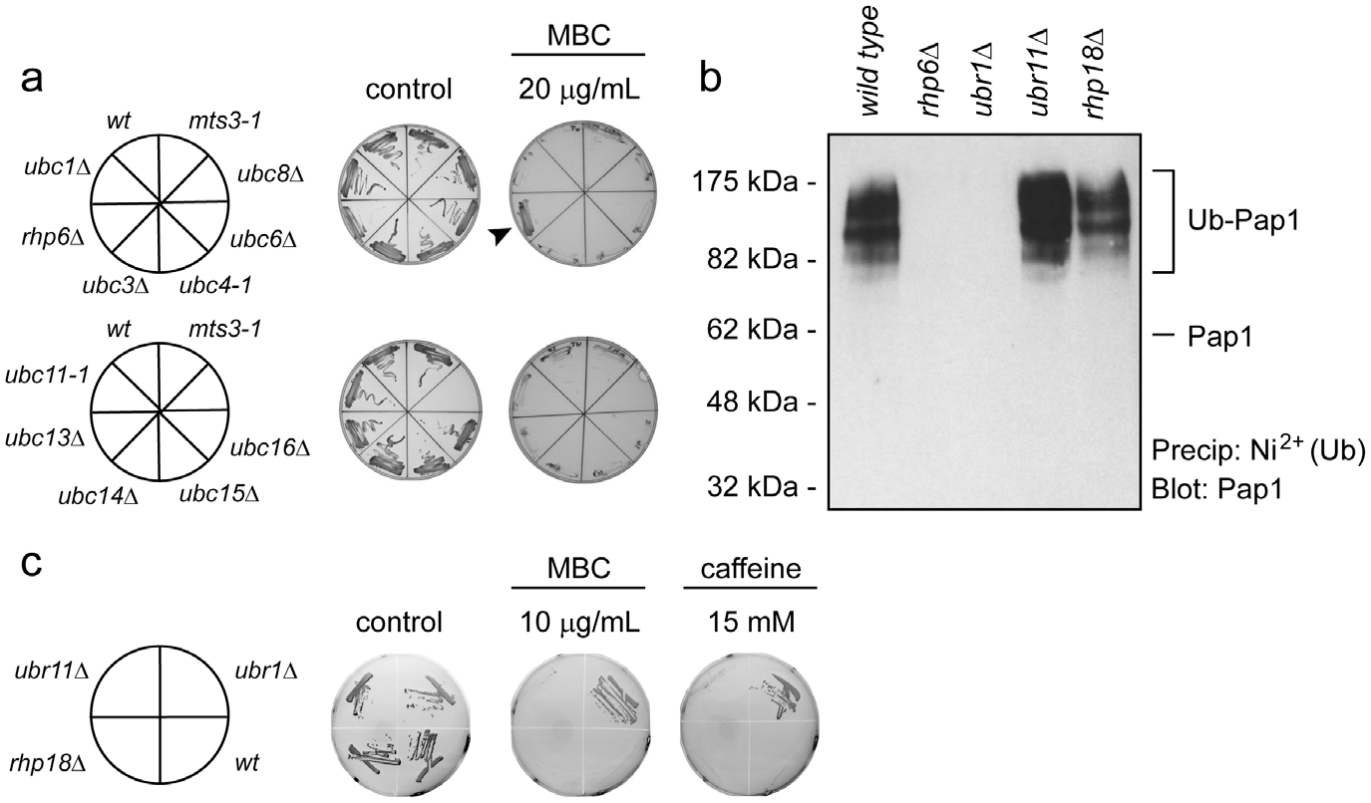
Pap1 is ubiquitylated by Rhp6/Ubc2 and Ubr1. (a) To identify which E2 ubiquitin conjugating enzyme is responsible for ubiquitylating Pap1, the indicated E2 null mutants were streaked onto solid medium containing MBC and grown for 48 hours at room temperature. Since the *rhp6*Δ cells grow in the presence of MBC (arrowhead) it was a candidate E2 in Pap1 ubiquitylation. (b) To identify the E2 and E3 enzymes responsible for ubiquitylating Pap1, the indicated strains were transformed to express 6His-tagged ubiquitin. The 6His-tagged ubiquitin was then precipitated using Ni^2+^ agarose under denaturing conditions and analyzed by Western blotting using antibodies to Pap1. Ubiquitylated species of Pap1 were detected in wild type, *ubr11*Δ and *rhp18*Δ cells, but not in cells lacking the E2 ubiquitin conjugating enzyme Rhp6 and in cells lacking the E3 ubiquitin-protein ligase Ubr1. (c) The indicated yeast strains (left panel) were streaked onto solid medium containing MBC or caffeine at the shown concentrations and incubated for 48 hours at room temperature. On the control medium lacking drugs all the strains grew. In the presence of drugs the growth of wild type cells was compromised, while the *ubr1*Δ mutant displayed resistance.

Ubiquitin is added to substrate proteins by the action of an E1, E2 and E3 enzyme cascade. We were interested in investigating which E2s and E3s were involved in the addition of the ubiquitin chain to Pap1. We postulated that the relevant E2 involved in targeting Pap1 protein for ubiquitylation and degradation should show resistance to MBC in an analogous manner to the *mts* proteasome mutants. The fission yeast genome encodes several different ubiquitin-specific E2 proteins. We obtained 8 null or conditional mutants that were generated previously (*ubc1, ubc2, ubc3, ubc4, ubc6, ubc11, ubc13* and *ubc15*), and constructed three additional null mutants (*ubc8, ubc14* and *ubc16)* for this study. The ORFs of *ubc8^+^* and *ubc14^+^* were replaced with the *ura4^+^* gene, while the *ubc16^+^* ORF was replaced with the hygromycin resistance gene. When these strains were streaked on plates containing MBC, only the mutant in *ubc2*, also known as *rhp6*, displayed MBC resistance (Fig. 5a), suggesting that Rhp6 is involved in Pap1 ubiquitylation.

To verify that Rhp6 is involved in Pap1 ubiquitylation, the expression plasmid for 6His-tagged ubiquitin was introduced in the *rhp6* null strain. Indeed Pap1 was no longer ubiquitylated in the *rhp6*Δ mutant (Fig. 5b). These data are consistent with the observation that the *rhp6*Δ mutant is the only E2 mutant of those tested to show MBC resistance and leads to the conclusion that Rhp6 functions as the major, or perhaps sole, E2 conjugating enzyme for ubiquitylation of Pap1.

### The Ubr1 E3 Ubiquitin-protein Ligase Targets Pap1 for Degradation

Since the budding yeast Rhp6 ortholog, Rad6, has previously been shown to interact with the E3 ubiquitin ligases called Ubr1 [22], Ubr2 [23] and Rad18 [24], we reasoned that the fission yeast orthologs Ubr1, Ubr11 and Rhp18, respectively, are likely candidates as E3s for ubiquitylation of Pap1. We therefore transformed *ubr1*Δ, *ubr11*Δ and *rhp18*Δ mutants with the expression plasmid for 6His-tagged ubiquitin and purified ubiquitin-conjugates as above. The results showed that Pap1 remained ubiquitylated in the *rhp18* and *ubr11* null strains, whereas no ubiquitylated Pap1 was detected in the *ubr1*Δ strain (Fig. 5b). This suggests that Ubr1, but not Ubr11 and Rhp18, functions as E3 ubiquitin-protein ligase in targeting Pap1 for degradation. Accordingly, we found that *ubr1*Δ cells are resistant to MBC and caffeine, whereas the *rhp18* and *ubr11* null strains are not (Fig. 5c). Collectively, this leads to the conclusion that Pap1 ubiquitylation and subsequent degradation is catalyzed by the Rhp6 E2 ubiquitin conjugating enzyme and the Ubr1 E3 ubiquitin-protein ligase. Moreover, the complete lack of ubiquitylated Pap1 in the *ubr1*Δ strain implies that the Ubr1 protein plays a major role in targeting the Pap1 protein for degradation.

## Discussion

Here we have described a genetic screen for mutants in fission yeast that showed resistance to the mitotic poison MBC that were also temperature sensitive for growth. Mutants in ten different essential genes were isolated. Nine of the genes encode different subunits of the 26S proteasome, while one encoded the nuclear export factor Crm1. The proteasome mutants were obtained in all the different subcomplexes which make up the 26S proteasome. Four mutants, *mts6*, *mts7*, *mts8* and *mts9* were in subunits of the 20S catalytic complex. Two mutants, *mts2* and *mts4,* were in subunits of the 19S regulatory base subcomplex. The *mts2* strain had mutations in one of the ATPase subunits (Rpt2) while *mts4* had mutations in the Rpn1 nonATPase subunit. The remaining three *mts* genes *mts1, mts3* and *mts5* encoded different subunits of the 19S regulatory lid sub-complex. All the *mts* mutants were conditional alleles of essential genes. Curiously, budding yeast null mutants in *RPN9* are viable [25]. We found that in *S. pombe* the *RPN9* ortholog *mts1* ^+^ is an essential gene. The isolation of mutants in all the different sub-complexes of the 26S proteasome would seem to indicate that a general defect in 26S proteasome function was responsible for the MBC drug resistance seen at the permissive temperature. Previous studies in budding yeast have also noted that mutants in proteasome assembly factors are resistant to alkylating agents [26], presumably this occurs via a similar mechanism as the one described here. In this paper we describe the mechanism which is responsible for the observed drug resistance. The Pap1 stress-activated transcription factor appears, by genetic and biochemical criteria, to be the sole, or at least primary, target for the different *mts* proteasome mutants that results in the drug resistant phenotype. In the *mts* proteasome mutants Pap1 protein is stabilized, resulting in increased Pap1-dependent activity and the observed multi-drug resistant phenotype. Up regulation of the Pap1 transcription factor has been implicated in the multi-drug resistant phenotype in a number of different screens, probably due to the upregulation of ABC transporters such as Bfr1 [27] [15] [28] that mediate drug efflux [13] [28].

The Pap1 protein is known to be tightly regulated at many different levels. Thus, Pap1 is activated by oxidation, but is also regulated on the level of its subcellular localization. In addition, the Pap1 protein is rapidly turned over by the UPS [29]–[31]. Hence, under non stressed conditions Pap1 is and located in the cytosol, while stress conditions trigger its nuclear translocation. However, as we show here, this regulation requires that the Pap1 levels are kept balanced by the UPS.

We propose that the fission yeast Rhp6/Ubc2 functions as the major E2 ubiquitin conjugation enzyme for Pap1 degradation, while Ubr1, but not the related Ubr11 and Rhp18, functions as E3 ubiquitin-protein ligase in targeting Pap1 for degradation. These data are in perfect agreement with recent results showing that Ubr1 regulates the fission yeast oxidative stress response by targeting Pap1 for degradation [31]. Intriguingly, the budding yeast orthologues of Rhp6 and Ubr1, called Rad6 and Ubr2, respectively, were found to regulate degradation of Rpn4, a transcription factor driving expression of most proteasome components [32]. Since Pap1 and Rpn4 are not related, this is most likely coincidental. Accordingly, in *Saccharomyces cerevisiae* degradation of the Pap1 orthologue, Yap1, was recently shown to depend on another ligase called Not4 [33].

Since budding yeast Yap1 transactivates expression of Rpn4 [34], it is possible that in the fission yeast *mts* mutants the impaired degradation of Pap1 leads to an increase in proteasome expression. If this is indeed the case, such a mechanism would only blunt the response we observe here. Moreover the existence of such a regulatory mechanism is not obvious, since the *S. pombe* genome does not encode any obvious orthologue of budding yeast Rpn4, the closest relative being Rsv2 that induces stress-related genes during spore formation [35]. Therefore it is currently unclear how proteasome gene expression is regulated in *S. pombe*. However, previous studies have shown that proteasome expression does not depend on Pap1 [36]. Accordingly, we did not observe any changes in proteasome levels in a *pap1*Δ strain (Fig. S1).

Originally Ubr1 was shown to be required for degradation of proteins carrying destabilizing residues in their N-terminus via the so-called N-end rule [37]–[38]. However, Ubr1 also recognizes misfolded proteins [39]–[40] and substrates carrying internal degradation signals [41]. More recently, Ubr1 was also linked to the Johanson-Blizzard syndrome [42], an autosomal recessive disorder that involves pancreatic dysfunction and mental retardation, that has been suggested to be connected with impaired transcription factor degradation [41].

Since all the components identified here (Pap1, Rhp6, Ubr1 and the 26S proteasome) are known to have orthologs in mammalian cells, it is likely that multi-drug resistance could occur by a similar mechanism in mammalian cells. Interestingly, it has been reported that the mammalian Pap1 ortholog, the c-Fos transcription factor, is targeted for degradation by the Ubr1 ortholog in human cells [41]. Thus, interference with this pathway, either genetically or by inhibitors, like bortezomib (Velcade), used in cancer therapy [43], may increase drug and/or stress tolerance also in mammalian cells.

## Supporting Information

Figure S1 **Proteasome levels are unchanged in a pap1Δ mutant.** Whole cell extracts from wild type (wt) and *pap1*Δ strains were analyzed by SDS-PAGE and Western blotting using antibodies to the 26S proteasome subunit Rpn1/Mts4 and 20S α subunits. Antibodies to tubulin were used to ensure an even loading. No significant differences between the strains in proteasome levels were observed.
(TIF)
