# Supplementary figures and images for: Fission Yeast 26S Proteasome Mutants Are Multi-Drug Resistant Due to Stabilization of the Pap1 Transcription Factor
Source: PLoS One. 2012 Nov 27;7(11):e50796. doi: 10.1371/journal.pone.0050796 (PMC3507774; doi:10.1371/journal.pone.0050796)

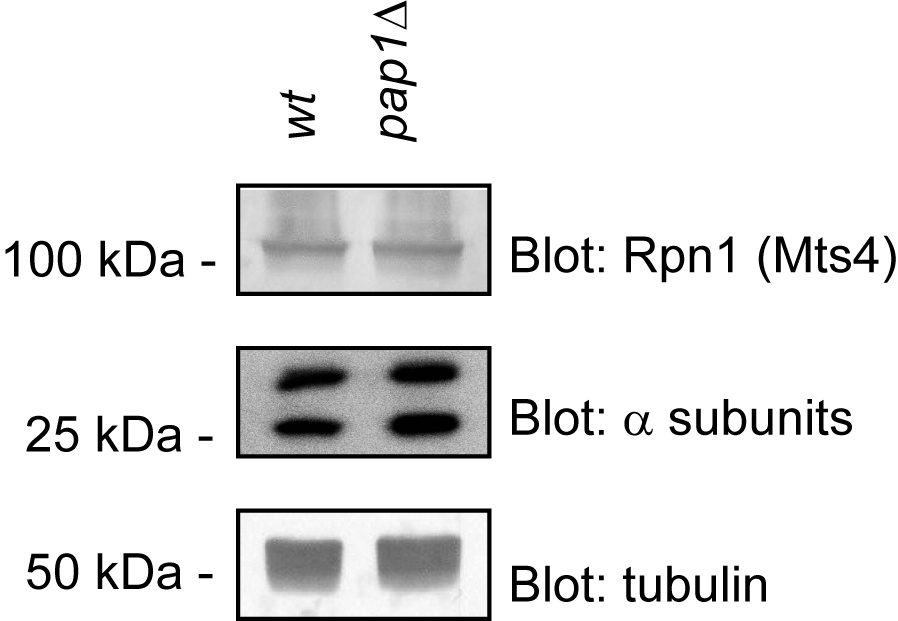

Supplement: Figure S1 — Proteasome levels are unchanged in a pap1Δ mutant. Whole cell extracts from wild type (wt) and pap1Δ strains were analyzed by SDS-PAGE and Western blotting using antibodies to the 26S proteasome subunit Rpn1/Mts4 and 20S α subunits. Antibodies to tubulin were used to ensure an even loading. No significant differences between the strains in proteasome levels were observed. (TIF) [file pone.0050796.s001.tif]
